# Attitudes of East Tennessee residents towards general and pertussis vaccination: a qualitative study

**DOI:** 10.1186/s12889-021-10465-w

**Published:** 2021-03-05

**Authors:** Corinne B. Tandy, Jennifer M. Jabson Tree

**Affiliations:** 1 Department of Biomedical and Diagnostic Sciences, The University of Tennessee, 2407 River Drive, Knoxville, TN 37996 USA; 2 Department of Public Health, The University of Tennessee, 367 HPER, 1914 Andy Holt Avenue, Knoxville, TN 37996 USA

**Keywords:** Immunization, Vaccination, Vaccination attitudes, Qualitative, Vaccine hesitancy, Pertussis

## Abstract

**Background:**

Despite vaccination being one of the safest and most successful public health tools to control infectious diseases, some people still doubt the efficacy and safety of vaccines. In order to address vaccine hesitancy and anti-vaccination sentiment, it is necessary to understand vaccination attitude development and vaccination behaviors. The objective of this project was to qualitatively investigate general vaccination attitudes and behavior with an additional emphasis on pertussis vaccination.

**Methods:**

To identify factors that influence attitudes toward vaccination and behaviors in East Tennessee, eleven one-on-one interviews were conducted with participants recruited through convenience and purposive sampling. Interview protocol and deductive codes were developed using the Triadic Theory of Influence as a theoretical framework. Interview transcripts were analyzed qualitatively and themes were identified through constant comparison of interviews, considering both deductively and inductively coded data.

**Results:**

Most participants (8) held positive attitudes towards vaccination. Participants (8) comfortable with vaccinating themselves or their children said they followed recommendations of doctors. Vaccine hesitant participants’ (3) most frequently cited concern was safety and concern about side effects. These participants also reported that they referenced non-academic or professional sources and felt confident about their knowledge of vaccines and diseases. Vaccine hesitant participants had low perception of risk of vaccine-preventable diseases, particularly pertussis. Participants with children reported that friends and family were influential when deciding to vaccinate their children.

**Conclusions:**

This study identified themes in the attitudes towards vaccination of participants recruited in East Tennessee. We found that risk perception and family and social group attitudes were the primary influences on vaccination decision making. We recommend that future research includes anti-vaccination participants in their research, if possible, and further explore the relationship between perception of one’s own knowledge and health behavior outcomes.

**Supplementary Information:**

The online version contains supplementary material available at 10.1186/s12889-021-10465-w.

## Background

The United States and Europe are experiencing a return of vaccine-preventable diseases which has been attributed to anti-vaccine movements [1]. The United States has experienced multiple measles outbreaks among mostly unvaccinated persons. Additionally, in 2018, the United States had one of the worst influenza seasons in decades; it is estimated that 80,000 people died, including many children who were not vaccinated despite ACIP recommendations [1].

Vaccines are one of the safest and most successful tools to control and prevent infectious diseases [2]. Despite this public health and medical truth evidenced by extensive empirical literature [3], doubt of vaccine efficacy as well as concerns over side effects by the general public has increased and has resulted in considerably lower vaccination coverage [2]. This rise in anti-vaccine sentiment has resulted in the World Health Organization (WHO) declaring vaccine hesitancy, defined as “the reluctance or refusal to vaccinate despite the availability of vaccines”, as one of the top ten threats to global health in 2019 [4]. Efforts to understand the contributors to and the context within people make decisions about vaccinations is necessary for understanding how to address vaccine hesitancy.

### Vaccination attitudes

Immunization attitudes are complex and influenced by social and cultural norms, family and peer attitudes, relationship with the medical community, and personal history of immunization experiences, amongst others. The most commonly cited reason for receiving vaccines given by vaccine supporters, is that they are recommended by healthcare professionals [5]. However, fear of vaccine-induced illness and negative side-effects has become a major barrier to vaccine acceptance and adherence [5–7], and there is evidence that vaccine safety is a major concern of parents when deciding to vaccinate their children [8–11]. Vaccine safety has also been identified as the most important consideration when making vaccine decisions [9–11]. According to Yaqub and colleagues’ (2014) critical analysis ‘safety concerns’ were the most frequently cited reason for hesitancy amongst both the general population as well as healthcare practitioners in the United Kingdom.

Recently public concern regarding adverse outcomes of vaccination such as autism and Guillain-Barré syndrome has risen [7, 12, 13]. Concern regarding autism as an adverse outcome after vaccination rose after Wakefield et al.’s 1998 study investigating the relationship between the measles, mumps, and rubella (MMR) vaccine and autism and diarrhea [14]. Despite a complete lack of confirmation by large epidemiologic studies [15], a retraction of the article on the grounds of research misconduct [16], and the scientific consensus being that there is no causal relationship between the MMR vaccine and autism, public concern and controversy about the risk for autism after vaccination has persisted. While concerns about safety and side effects, particularly those discussed here, are among the most commonly given reason for vaccine hesitancy, other factors such a general distrust of the medical community [17], the government agencies recommending the vaccines [18], and a growing interest in treating disease with homeopathic treatments and obtaining “natural” immunity through exposure to the disease, contribute to hesitancy towards and rejection of vaccines [19].

### Attitudes towards pertussis-containing vaccines

Lack of awareness or lack of perception of need has been established as a major barrier to pertussis-containing vaccine receipt. We do not know, however, what and if other health behavior, attitudinal, and decision-making factors play a role in people’s decision regarding pertussis vaccination. Filling this gap is necessary as the incidence of pertussis is increasing and can cause severe illness in one of our most vulnerable populations—infants.”

### Approaches to understanding health behaviors and vaccine attitudes

Several models of health behavior have at their foundation the assumption that health behavior decisions are made rationally (e.g. Health Belief Model, Theory of Reasoned Behavior). However, we know that people often make health decisions that are not in their best interest or rationally. Behavioral economics asserts that people do not act rationally, make decisions based on, amongst other things, inadequate understanding of possible outcomes, and do not always learn from their mistakes [20]. Because of this, it is necessary to consider psychological and sociological influences when seeking to understand health behavior. We sought to explore vaccination attitudes using a theoretical model that does not assume rational decision making in health behavior, specifically the Theory of Triadic Influence (TTI).

Understanding the social and psychological contexts in which people develop their vaccination attitudes and make vaccination decisions is necessary to address vaccine hesitancy [8]. The Theory of Triadic Influence (TTI), an integrative, ecological model, assesses the proximal and distal influences on behavior [21]. Extant literature suggests that individual rights [22], previous medical experiences [23], risk assessment [24], and family and friends’ attitudes towards vaccination [5] influence how people make their vaccine decisions. The TTI includes perceived sense of control, motivation to comply, others’ behaviors and attitudes, perceived norms, knowledge, information sources, and institutional interactions as central concepts. We used the TTI as a framework to investigate how these components related to attitudes and behaviors regarding vaccination.

Understanding vaccination attitudes is critical to understanding vaccine hesitancy, which has been identified as a major barrier to vaccination coverage. In fact, a call to action regarding further understanding of attitudes towards vaccinations and the barriers hesitancy and negative attitudes present has been made by global research and public health communities [4, 25]. The goals of this project were to qualitatively investigate vaccine attitudes and behavior, with emphasis on pertussis vaccination. We sought to explore these phenomena by developing a semi-structured interview protocol based on an integrative, ecological behavioral model.

## Methods

### Participants and recruitment

This study involved two samples. The first sample of this study was drawn from the population of the greater Knoxville, TN area. Participants were recruited using convenience sampling from community events and through distribution of advertisements around the greater metro area. Participants were recruited from sixteen community events that occurred between June 2016 and July 2018. Events included farmer’s markets and multicultural events from downtown, northern, and western areas of the greater Knoxville area. Flyers were distributed at these events to recruit people to participate in the study, which led to eleven interviews, all of which took place in-person at a private location of the participants’ choosing.

The second sample was purposely recruited using advertisements in a Facebook group for people in the Knoxville area that explicitly identifies as anti-vaccination. Between June 2016 and July 2018, a total of ten posts were made in this group to recruit participants. This purposive sampling yielded three responses and zero interviews.

We used snowball sampling with both samples. Participants from both samples and people who expressed an interest in this study were asked to aid in recruiting anyone that they knew who would be interested in participating. We considered that this approach [26] may increase participant response, particularly from the second, anti-vaccination sample where trust of researchers is already low and positive word-of-mouth amongst the community may increase participation. Physical and/or digital copies of the recruitment flier were offered to all participants or interested persons.

Study participation was voluntary. To maintain confidentiality, participants’ first and surnames were not used; interviewees were prompted to select a pseudonym and if they had no preference a pseudonym was assigned to them. No incentives or remuneration were offered to participants.

### Procedures

The participants, prior to the beginning of the interview and after obtaining informed consent, completed a questionnaire [see additional file 1] developed by the authors for this study to collect demographic information. Demographic variables included: race, age, education level, gender, income, and health insurance status [27, 28].

“The study questionnaire asked whether or not the participant has ever received any vaccinations, if they have children, and if they vaccinate their children. It also asks if they have received a tetanus shot in the last ten years. If they responded “Yes”, they were asked if they knew if it was pertussis-containing or if they weren’t sure what type it was.”

“Semi-structured interviews were conducted with participants (*n* = 11). Semi-structured interviews followed our pre-determined interview guide [see additional file 2] that was developed by the first author with guidance, with supervision provided by the faculty second author. The interview guide consisted of questions and topics that would be covered in the interviews but also allowed for the interview to cover other topics if and as they arise [29]. Semi-structured interviews provided consistency across interviews while also providing flexibility for unanticipated responses and interviewer follow-up to responses.”

“Interview topics were developed based on previous literature and theory. Individual values [22], previous medical experiences [23], risk assessment [24], and family and friends’ attitudes towards vaccination [5] are particularly influential in the development of vaccination attitudes and decisions regarding vaccination. Our protocol included questions about past vaccination experiences, vaccine experiences regarding the participants’ children (if applicable), comfort level with the medical community, perceived vaccination attitudes of family and social networks, and the participants’ knowledge of pertussis.”

“The interview guide was informed by Theory of Triadic Influence (TTI) [21]; a framework which includes perceived sense of control, motivation to comply, others’ behaviors and attitudes, perceived norms, knowledge, information sources, and institutional interactions as central concepts. Our interview guide was developed to understand the participants’ thoughts about these concepts as they relate to vaccination. For example, the TTI suggests that others’ attitudes and behaviors influence perceived norms and motivation to comply, therefore we explicitly asked our participants about their family and friends’ attitudes towards vaccination. We asked follow-up questions regarding concerns expressed by friends or family to understand how participants’ attitudes and behaviors were received by family and friends. While other models exist that incorporate multiple areas of influence, such as the Vaccine Hesitancy Determinants Matrix, the TTI models the interactions between the streams of influence, adding a directionality that the VHDM does not incorporate. This directionality can inform the application of the model in practice and guide research questions as well as a framework for interpreting respondents’ opinions and responses.”

All interviews were conducted in person and were audio recorded with the consent of the participants. Interviews ranged from 30 to 75 min in length. Interviews were transcribed by the first author in word processing software.

This project was approved by the University of Tennessee Institutional Review Board (UTK IRB-15-02724-XP).

### Analysis

Qualitative transcripts were entered into NVivo 12 [30] for data management and analysis. Transcripts were read and reviewed multiple times to achieve immersion. Transcripts were analyzed continuously which allowed for consideration of saturation defined as no new themes emerging from additional transcripts [31].

Saturation was applied as a guide for sample size. Following the definition by Given (2016), that additional data do not lead to any new emergent themes [32] . We knew that we had arrived at saturation when, when coding additional data no new codes occurred and mounting instances of existing codes occurred instead [33].

A hybrid approach of theory-informed and data-driven deductive and inductive coding was used when analyzing interview data. For deductive coding, a priori codes were developed based on the Theory of Triadic Influence as well as extant literature [34]. The first cycle of coding, deductive coding, included nine codes, seven of which were developed utilizing the Theory of Triadic Influence [35] (Table 1). These codes were developed to identify anticipated responses based on the Theory of Triadic Influence as well as by prior literature, including academic and media publications about vaccination attitudes and behaviors. Theory driven codes were based on the distal predisposing influences and proximal immediate predictors of the TTI model and indicated with an asterisk in Table 1.

**Table 1:** Deductive and Inductive Codes Utilized in Qualitative Analysis

Deductive Codes	Comfort with medical community Concerns about vaccines Family beliefs^a^ Social group beliefs^a^ General attitude toward vaccines^a^ Risk perception^a^ Related behaviors^a^ Knowledge of disease^a^ Information sources^a^ Reason for vaccinating children Reason for not vaccinating children	
Inductive Codes	Confidence in Science Comfort Common Sense Evidence Too many vaccines Research Experience resulted in change Making own choices/individual freedoms Trust	
Theme 1 “Attitudes Toward Vaccination” Codes	General Attitude: Evaluation: Positive	“I think that [vaccination] is a good idea”; “I generally think it is a good thing. It is saving lives and protecting people, stopping diseases from being passed around”
	General Attitude: Evaluation: Negative	“The flu shot, that’s just a load of crap”; “I think some vaccines work”
Theme 2: “Reasons for Supporting Vaccination: Codes	Reason for Vaccinating Children	“Because children used to die because we had all these diseases”; “make sure they don’t get these common diseases, protect them in the future”; “everybody gets
Theme 3: “Concerns and Reasons for Vaccine Hesitancy” Codes	Perceived Risk	“I know the flu shot is recommended but I have not caught the flu in a long time”
	Perceived Risk of Getting Pertussis	“now that the kids are older, I don’t see how we could get [pertussis]”
	Concerns about Vaccines	“Maybe like allergic reactions”; “I’m more of a skeptic about what the government says is safe”; “The idea that mercury is one of the preservatives”
	Too Many Vaccines	“I think if you pump five vaccines into your child, their poor little bodies can’t deal with it”
Theme 4: “Types of Knowledge and Information Sources” Codes	Knowledge—Information Sources	“Mostly just our doctors”; “Our own research”; “WebMD”

^a^TTI informed codes

Inductive codes (Table 1) were identified throughout the coding process as unanticipated themes or responses were observed in the transcripts during reading and coding. Inductive coding was informed by the enumeration of terms and phrases and constant comparison methods [36]. These codes described a new theme or expanded on a priori deductive codes. See Table 1 for a complete list of codes.

Four predominant themes emerged from the data: Attitudes Toward Vaccination, Reasons for Supporting Vaccination, Concerns and Reasons for Vaccine Hesitancy, and Types of Knowledge and Information Sources. These themes were identified through constant comparison of interviews, considering both deductively and inductively coded data.

## Results

### Participant demographic characteristics

Participant’s demographic characteristics are summarized in Table 2. Most participants (*n* = 10, 91%) self-identified as white and non-Hispanic, all participants had completed high school and most had completed college (*n* = 7, 64%). Most of the study participants (*n* = 9, 81%) had health insurance and the majority (*n* = 6, 55%) had children. The mean age of the participants was 34 years and the majority were female (n = 6, 55%).

**Table 2:** Demographics of Participants

Age (mean)	34
**Gender**	
Male	45%
Female	55%
**Race/Ethnicity**	
White	91%
Black	0%
Hispanic	9%
Asian	0%
**Education**	
High School or Higher	100%
College or Higher	82%
Graduate School/Professional	64%
**Annual Household Income**	
< 10,000	9%
10,001-25,000	18%
25,001-40,000	9%
40,001-60,000	27%
60,001-75,000	27%
> 75,000	0%
**Health Insurance**	
Yes	81%
*Private*	45%
*Public*	36%
No	9%
**Vaccinated?**	100%
Tetanus	
*Yes*	82%
*Unsure*	18%
Tdap	
*Yes*	27%
*Unsure*	73%
**Has Children**	55%
**Children Vaccinated**	100%
**Children have DTaP**	100%

### Theme 1: attitudes towards vaccination

The majority of respondents (*n* = 8, 73%) held positive attitudes regarding vaccination in general. If participants had had children, they reported that they had their children vaccinated and up-to-date according to current recommendations. Expressions of support ranged from vague positivity to explicit and thorough sentiment. For example, Aileen said that they “think [vaccination] is generally a good thing,” and Amelia reported that they “think vaccinations are a great thing,” and even considered getting vaccinated part of their civil duty. They continued, “I think that in order to keep people healthy that is basically a civil … part of being a citizen is to have your children vaccinated …”.

Three (27%) participants had either mixed or negative feelings about at least some aspect of vaccination. Vaguely negative sentiments expressed included Fred indicating that they perceived the necessity of vaccines differently by age group, stating “I think some vaccines work. Obviously, the ones for the polio, the ones you receive as kids. I think those work and flu vaccines are needed maybe for children and the elderly but not as a yearly thing.” Explicitly negative sentiments included participants doubting the efficacy of some vaccine completely; as Rosemary said, “The flu shot … that’s just a load of crap.”

### Theme 2: reasons for supporting vaccination

The most commonly offered explanation when asked why parents chose to vaccinate their children was to prevent their children from getting diseases. “Because we don’t want [them] to get sick,” shared David. Most participants that held positive beliefs about vaccination also cited historical prevalence of many diseases as a contributing factor to their consideration of vaccination. Aileen shared that one of their major considerations when considering vaccination as a general practice was the availability of modern technology and medicine to prevent diseases,

Because children used to die because we had all of these diseases that killed off I don’t know how many children in their early years and I think it is incredible that we have these things where you can have one shot and then we don’t have to worry that our kids are going to die of all these … I mean it makes me want to cry that I don’t’ have to worry about it. So I just think it of one of those achievements of modern medicine that we can eradicate and prevent some really awful things.

Others shared that they would feel guilty if their child got someone else sick. David expressed concerned that they “wouldn’t want [their child] to be the vector that got one of [their] immune-suppressed friends sick.”

### Theme three: concerns and reasons for hesitancy

#### Number of vaccines administered

Vaccine-hesitant participants cited side effects and the number of vaccines administered at once as the primary reasons for delaying their children’s vaccination schedule. Aileen said that they felt like “it’s a lot of stuff to put in [their] tiny body in one visit.” Fred and Rosemary also expressed the same concern. “Personally, I think if you pump five vaccines into your child, their poor little bodies can’t deal with it,” Rosemary offered. Fred asked “How much can your auto-immune system take? Is that something that you should really be pushing with your children’s health?”

#### Denial of efficacy and safety concerns

Some participants reported that a major contributor to their refusal or hesitancy to get vaccines included not trusting vaccine efficacy. “The flu shot … that’s just a load of crap,” shared Fred. “Like, you’re injecting the flu shot into your body. It’s a virus … you can’t kill it. So your chances of getting the flu when you take the shot are higher than when you don’t.” Not only does this reject that the influenza vaccine is effective it also suggests that the vaccine causes the very illness it is intended to prevent.

Fred was the only participant to explicitly reject vaccines as effective.

#### Concern regarding safety, side effects, or negative consequences of vaccination

Most (*n* = 9, 82%) participants expressed minimal concern regarding the safety of vaccines and stated that while they had safety concerns, they were not significant enough to cause them to delay or deny vaccines for themselves or their children. David reported that when they were getting their first child vaccinated they were concerned that their child would have an allergic reaction or adverse outcome because of the ingredients. “Oh there’s egg albumin, does she have any allergies? Then the idea that mercury is one of the preservatives … ,” David recalled from when they had their first child and said that their concern as normal, explaining “Oh, we’re parents! We worry.”

Some participants had major concerns about side effects and other adverse outcomes. When discussing vaccines and particularly the influenza vaccine with Rosemary, they expressed major concerns about the vaccine’s safety and its impact. “The thing that I worry about, really, is these diseases mutating and making a comeback … kind of like how the flu makes a new strand every time they come out with a new vaccine.” Fred added,

I don’t think [influenza vaccines] are healthy for adults. The RNA mutation is a big concern. And they say that the influenza in the shot is dead but I’m like ‘yeah but how do you know that?’ That’s what I can’t seem to get an answer for. A virus can reanimate at any time and you can’t guarantee that. Other than that, I think the rest of them are safe.

#### Risk perception of getting pertussis

Most participants conceptualized risk of disease differently for their children than they did for themselves. In fact, many reported that their children were fully vaccinated against pertussis, but they themselves were not sure if they were up to date. Aileen seemed to become aware of this incongruence between their behaviors and self-described attitudes while discussing risk in adult and children. “I feel in some ways that my attitudes are more pro than my actions,” they said while discussing them not receiving an influenza vaccine this year while their children did. They went on to say that “maybe I have some kind of division in my mind between children and adults.”

All participants perceived themselves at very low risk for contracting pertussis. Fred thought that “now that the kids are older, I don’t see how we could get it.” Rosemary stated, “I don’t think we can get it. The doctor says everyone carries it around but from what I’ve been told, the chances of an adult getting it are slimmer than elderly or an infant so we’re not worried.” Other notable responses included “I feel like I’ve heard that it effects a lot of elderly people, maybe? That’s about it,” offered Ted. Nancy thought they didn’t “think [they] could get it, because [they’ve] had the vaccine.” “I hope,” they continued, “And if I did get it, it probably wouldn’t be as serious.”

#### Theme four: knowledge and information sources

The majority of participants reported speaking with physicians or another healthcare provider about vaccines when prompted, but otherwise reported either not intentionally seeking information or seeking it on the internet. Participants that reported never having sought information themselves reported that they spoke with their doctor when it was necessary.

Conversations with doctors, pamphlets at physicians’ offices, and internet research were the most cited sources of information about vaccinations. Elizabeth stated that they talked to “my doctor and my kids’ doctors. My husband a little bit. And I’ve heard stories on the news or heard things on the radio, people on TV.” They admitted “Sometimes I google things about ‘what is this … ’ but generally I get more information when I go to the doctor and they hand you a sheet that has pros and cons and possible reactions …” .

Amelia reported that they consulted their “primary care physician” and got most of their information at the “the doctor’s office. They give you those pamphlets.” Though the same participant also said, “I’ll also go online and look up things … I’ll google and then go to maybe Mayo Clinic, or someplace I recognize … Cleveland Clinic, CDC, some place I feel is more credible.”

Participants who perceived their knowledge of pertussis and vaccination as high did not have high knowledge of these topics and referenced popular science writing as their primary information sources. Fred and Rosemary cited vague sources like “medical books from the library” and popular science blogs including “IFL Science” as sources of information.

Many parents that participated in this study (*n* = 6, 55%) reported that they did not do much research about vaccinations or side effects until faced with the idea of vaccinating their children. Aileen said that they went through “some kind of evolution as far as how I felt about it because I didn’t do a ton of research before I had kids.”

Outside of physicians and the internet, participants reported discussing vaccinations with their family and friends and other social entities. Casey even cited “physicians and coworkers” as the primary two entities with whom they discussed vaccines. David said they talked with their friends and spouse about vaccines. They report discussing people in their larger social network that plan to not vaccinate their children and the participant reports that they have conversations about that so that they can try to understand. Fred reported discussing vaccines with their mother who is “a nurse and she even did a little paper about the chemicals in the vaccines and the things that build up in your system.”

## Discussion

We found that our participants were generally supportive of vaccination, though some expressed hesitation and concerns. Two participants would be best described as “hesitant compliers”, people who have major concerns or objections but ultimately decide to comply with vaccination recommendations. Knowledge about vaccines and disease risk, types of information sources utilized, and the attitudes of friends and family were important considerations in our participants’ vaccine behaviors.

### Dunning-Kruger, knowledge, and vaccine hesitancy

An important finding in our study was that participants that perceived their knowledge of pertussis and vaccination as high did not, in fact, have high knowledge of those topics. This cognitive phenomenon is known as the Dunning-Kruger effect [37]. These participants were more likely to cite non-academic, non-peer-reviewed, or vague sources of information when attributing facts. Our participants who demonstrated the most confidence in their knowledge of pertussis and vaccination and their associated risks were also the most likely to be incorrect about pertussis risk factors and vaccination side effects. These participants were also the most vaccine hesitant. Motta and colleagues (2018) propose that vaccine receipt and vaccination policy ideas are influenced by the Dunning-Kruger effect, a cognitive bias that results from an overconfidence and assessment of their own knowledge regarding a subject as higher than it truly is [37]. Fred is an excellent example of this phenomenon—they offered what they considered factual knowledge about vaccinations and pertussis with confidence (“The flu makes a new strand every time they come out with a new vaccine”), referred to the information sources they utilized and thought them trustworthy (“IFL Science and New Scientist … I try to go with more credible sources.”), and had major concerns about vaccinating their children (“How much can your auto-immune system take? Is that something that you should really be pushing with your children’s’ health?”). Fred’s overconfidence in their own knowledge of pertussis, vaccination, and biology is not unusual in vaccine-hesitant or vaccine-refusing parents [38].

Participants that shared concerns about side effects or consequences of vaccination but who were not as confident in their knowledge reported following the professional recommendations of the medical community. David admitted that while they “didn’t know much about vaccines,” and “were more on the disbelief side and with the belief that there might be some link to autism,” they ultimately vaccinated their children at their physician’s recommendation. Vaccinating despite being uncertain about health or other outcomes is common in vaccine-hesitant parents [39].

Finally, participants that admitted their lack of knowledge regarding pertussis or vaccination, such as Casey, who at one point in our conversation asked, “is there a vaccine for pertussis?”, were fully vaccinated and did not express any concerns that would prevent them from getting a vaccine.

This spectrum of knowledge confidence and outcomes seen in our participants echoes the hypothesis that overconfidence in one’s own knowledge about vaccines is consistent with high hesitancy [40].

While the vaccine hesitancy expressed by participants in our study revolved around confidence in inaccurate knowledge, lack of knowledge, concern over side-effects, and lack of awareness, work from international studies highlights the wide variety of reasons that contribute to vaccine hesitancy. Research conducted by Hobson-West et al. [41] and Rozbroj et al. [42] identified that British and Australian respondents name lack of trust in the government and medicine, as well as the desire to make individual vaccine-related choices, as prominent themes in vaccine hesitant and refusing people. The importance of individual decision making in vaccine receipt was also highlighted in a recent Australian study; Rozbroj et al. (2019) characterized people with negative attitudes about vaccination as “engaged health consumers” who considered it risky to blindly trust health officials [42]. This same lack of trust in health officials has been noted in a British study of vaccine hesitance and refusal [41]. Interestingly, in the same study, researchers identified the desire to make individual judgements of vaccine safety and subsequent health decisions with personal health empowerment [41]. It is clear that individual decision-making and empowerment are internationally prominent themes in vaccine-related behaviors. In addition, there is evidence of significant variation in vaccine-related attitudes and behaviors by location. We submit that this underscores the need to be sensitive to local, regional, and national themes in vaccine hesitancy in order to advance successful educational and public health intervention strategies.

### Social and familial group influence

Many participants reported that family and friends significantly contributed to their vaccination beliefs and decisions to get a vaccine for themselves, particularly when considering vaccinating their children. These ideas are present beyond the United States and previous international literature suggests that social networks play a significant role in vaccination decision-making for parents [43]. Attwell et al. explored the beliefs of vaccine refusing parents in Australia through the lens of Social Identity Theory, focusing on how these parents frame their relationship with mainstream medical and parenting practices as critical to their identities and situate themselves socially. Participants in this study considered preventative vaccine behavior as the “Unhealthy Other”. Parents cited examples such as poor diet and overuse of pharmaceuticals as evidence that mainstream health practices, including vaccination, were damaging to their child’s health. These parents expressed that their holistic practices would replace the need for vaccination if they maintained good overall health by eliminating over the counter and prescription medications and instead focus on diet and nutrition. We also found evidence of the impact of social networks on vaccine related behaviors. Many participants in our study reported that family and friends significantly contributed to their vaccination beliefs and decisions to get a vaccine for themselves, especially when considering vaccinating their children. Rosemary and Fred mentioned that they got their kids their pertussis-containing vaccine because Fred’s mother “encouraged us to do it” and that “some friends that had kids said so.” Another participant reported that they had not considered not vaccinating their child until a family member suggested that they should not. Ours and others [44–46] highlight critical themes in vaccine-refusing groups and particularly that these groups self-perpetuate their anti-vaccination beliefs through social influence from in-group members, family and friends [44].

Anti-vaccination attitudes are not a primary social ideology in the United States. Studies conducted in the United Kingdom, France, and Australia, have demonstrated that people who hold anti-vaccination beliefs are small in number and often feel persecuted or unable to safely share their thoughts as well as feeling they are dismissed or marginalized [44, 45, 47, 48].

Unfortunately, we were unable to recruit any participants who reported being wholly anti-vaccination, despite purposive recruitment efforts. The purposive sampling methodology allowed us to access a large anti-vaccination group in the study area, but no members of the group agreed to participate. We expect that this is the result of social network characteristics and other cultural and ideological factors shared by this population. It is possible that the anti-vaccination groups in the United States, such as those in our study area, share a reluctance to participate due to feeling unsafe or fearing persecution as documented by Attwell et al. [44], Peretti-Watel et al. [45], Ward et al. [47], and Ward et al. [48]. Threat of negative social consequences associated with not vaccinating children, specifically concerns over child protective services being called, have been described by some, as a barrier to research participation by anti-vaccine parents in the United States.

### Pertussis vaccination

Our research did not identify any specific attitudinal barriers to pertussis vaccination beyond the extent empirical evidence concerning barriers to vaccination in general. A lack of awareness of the pertussis containing vaccine was common amongst our participants, and 73% of participants unsure if they were vaccinated against pertussis or not. Participants that were aware of their vaccination status against pertussis, volunteered that exposure to recent news reports, or recently having had a baby, as the primary reasons they knew they were vaccinated. Many participants perceived their risk of getting pertussis to be low, assumed they were vaccinated as children and therefore still protected. Others assumed that pertussis was only a health risk for infants.

Low perception of risk for pertussis was identified as the predominant factor in pertussis-containing vaccine refusal and hesitancy in adults across the United States [49]. 44.7% of respondents indicated that low risk of contracting pertussis was the reason that they did not get or refused to get the Tdap vaccine. Interestingly, most unvaccinated respondents (81.8%) in Miller et al.’s study indicated that they were willing to get the Tdap if it was recommended by a healthcare provider. These findings indicate the need to not only increase awareness and education about pertussis in adults and the role adults play in pertussis transmission to vulnerable populations but also to continue to emphasize the importance of healthcare provider recommendations in vaccine receipt.

Healthcare providers play a critical role in vaccine behaviors and hesitancy. Leask et al. recommend that medical education include training about vaccines themselves as well as the social dynamics of vaccination decision making and vaccine hesitancy experienced by patients [50]. Because healthcare providers play such a critical role in vaccine behaviors, Attwell et al. have proposed that providers who share common beliefs held by vaccine hesitant patients, particularly beliefs in non-Western approaches to health and holistic health approaches, could present an opportunity for vaccination education [44]. Additionally, training providers to discuss vaccine hesitancy in the context these common beliefs would benefit not only the providers in delivering preventive care but also the patients, as they may be more receptive to advise from providers whom they feel respect their health values.

The importance of healthcare provider recommendations is not unique to pertussis vaccination, but we propose that the degree to which people are unfamiliar with the disease is. Awareness of risk of illness or awareness of role in the disease transmission process is very low for pertussis. A recent study [51] investigating the association between vaccine refusal and vaccine preventable diseases, specifically focused on measles and pertussis in the United States, found that vaccine refusal was associated with measles vaccination than pertussis vaccination. The authors emphasize that vaccine refusal was still associated with increased pertussis risk in some populations even though pertussis resurgence has been at least partially attributed to waning immunity. Because the Tdap booster is critical to addressing waning immunity, increasing awareness of pertussis risk in adult populations appears necessary to increasing Tdap receipt.

### Strengths and limitations

This study has several limitations. We were unable to successfully recruit any explicitly anti-vaccine persons despite purposive recruitment strategies. As a result, those voices are not included in our study. Additionally, there is a distinct lack of racial/ethnic diversity in our participants. Although the study area is predominately white (> 75%), intentional efforts to understand the attitudes of the area population from different racial and ethnic backgrounds is recommended in future work. Because those voices are not sufficiently represented in our respondents, it is necessary to acknowledge that our findings come from a predominantly white population from varied economic backgrounds with relatively high education. Future research should include the perspective of people who hold anti-vaccination attitudes so that those may be better understood and so that we can develop and test interventions that would assist in improving vaccination rates for these groups. All participants were recruited directly, and snowball sampling yielded no additional respondents. Finally, the study sample for this research was small but saturation was achieved.

Despite this, the authors still feel confident that the major research goal of understanding important factors of vaccine attitudes was addressed. Additionally, the theoretical framework that was utilized to create the interview protocol and initial deductive coding in analysis aided in analyzing interplay of social, cultural and contextual factors of vaccine attitudes and behaviors. To the best of the authors’ knowledge, this study is the first to utilize the Theory of Triadic Influence to analyze vaccine attitudes and behavior at the time of this writing.

## Conclusions

The results of this study identified themes in the vaccination attitudes of persons in Knoxville, TN. We found that risk perception and family and social group attitudes were the primary influences on vaccination decision making, whether for themselves or on behalf of their children. This is consistent with prior literature that aims at increasing trust in and improving access to accurate knowledge about vaccination, increasing educational campaigns regarding vaccine-preventable diseases, and highlighting the importance of a physician or medical professional actively addressing vaccine hesitancy in clinical settings. Our results contribute to the knowledge base utilized by public health officials to develop and direct immunization health policies and initiatives, as knowledge of local attitudes and barriers towards vaccination are necessary to guide vaccination coverage improvement and awareness campaigns. We recommend that future research aims to include anti-vaccination participants in their research, if possible, as well as make efforts to further explore the relationship between perception of one’s own knowledge and health behavior outcomes.

## Supplementary Information

**Additional file 1.** Demographic Questionnaire.pdf; Questionnaire; this questionnaire collected demographic data about each participant.

**Additional file 2:.** Interview Guide for Article.docx, Interview guide, guide used for semi-structured interviews.

## Data Availability

Qualitative data not available for upload. Anonymized data may be available upon reasonable request with permission from IRB.

## Abbreviations

TTI: Theory of Triadic Influence
CDC: Centers for Disease Control and Prevention
Td: Tetanus and diphtheria
Tdap: Tetanus, diphtheria, and acellular pertussis
WHO: World Health Organization
ACIP: Advisory Committee on Immunization Practices
MMR: measles, mumps, and rubella
HPV: Human papillomavirus
VHDM: Vaccine Hesitancy Determinants Matrix
