# Supplementary material for: Attitudes of East Tennessee residents towards general and pertussis vaccination: a qualitative study
Source: BMC Public Health. 2021 Mar 5;21:446. doi: 10.1186/s12889-021-10465-w (PMC7934522; doi:10.1186/s12889-021-10465-w)
Supplement: Supplementary file 1 — Additional file 1. Demographic Questionnaire.pdf; Questionnaire; this questionnaire collected demographic data about each participant. [file 12889_2021_10465_MOESM1_ESM.pdf]

Date:____________
Participant Number ______

Demographic Questionnaire

Age: ____________

Gender (please circle): Male Female Transgender

Another Gender Identity (fill in blank if desired):_________________

Prefer not to answer

Race/Ethnicity (Circle all that apply): White (Non-Hispanic) Hispanic Black Other:_______________

Education (please circle):

Some High School
High School Degree
Some College
College Degree
Graduate/Professional Degree

Average Annual Income (please circle):

<10,000	10,001-25,000
25,001-40,000	40,001-60,000
60,001-75,000	>75,000

Do you have health insurance? (please circle): Yes No

If yes: Private Government

Have you ever received a vaccine? (please circle): Yes No

If yes, Have you gotten a tetanus shot in the last 10 years? **(please circle):** Yes No

If yes, do you know if it included protection against pertussis/whooping cough (Tdap)? **(please circle):**

Yes No Don't Know

Do you have children? (please circle): Yes No

If yes, have they ever received a vaccine? Yes No

If yes, have they received their pertussis/whooping cough (DTaP) series?**:** Yes No
