# Supplementary material for: Attitudes of East Tennessee residents towards general and pertussis vaccination: a qualitative study
Source: BMC Public Health. 2021 Mar 5;21:446. doi: 10.1186/s12889-021-10465-w (PMC7934522; doi:10.1186/s12889-021-10465-w)
Supplement: Supplementary file 2 — Additional file 2:. Interview Guide for Article.docx, Interview guide, guide used for semi-structured interviews. [file 12889_2021_10465_MOESM2_ESM.docx]

1. **Vaccine Attitudes and Knowledge**
   1. I’d like to hear your thoughts about vaccination in general.
      1. What positive things come to mind when you think about vaccination?
      2. What negative things come to mind when you think about vaccination?
   2. Who do you talk to about vaccines?
      1. What do you talk about when you talk about vaccines?
      2. Who do you feel most comfortable talking to about vaccines?
   3. How would you describe your family’s attitudes towards getting vaccines?
      1. What do they think about getting vaccines?
      2. What concerns have they expressed about getting any vaccines?
   4. How would you describe your friends’ attitudes towards getting vaccines?
      1. What do they think about getting vaccines?
      2. Have any of them ever expressed concern that you were getting any vaccines?
   5. Where do you get information about vaccines?
2. **Vaccination Behavior**

**[Self]**

- 1. From your survey it looks like that, at some point in your life, you have received a vaccine. Can you describe one of your experiences when you’ve received a vaccine?
  2. Tell me about a time that you have purposefully sought out a vaccine(s). For example: making an appointment specifically to get one?
     1. *Prompts and follow ups*:
        1. What were your reasons for getting that vaccine?
        2. What made you decide to schedule the appointment?
        3. What, if any, other options did you consider instead of vaccination?
  3. Where do you find it easiest to get your vaccines?
     1. What about this place makes it easier for you?
  4. Is there anything about getting a vaccine that would you change?
  5. What kind of unpleasant experiences have you had getting vaccinated?
     1. What caused the unpleasantness?
     2. How have negative experiences like that one influenced your thoughts about future vaccination?
  6. How do you feel about going to the doctor or talking to doctors?
  7. How has your doctor impacted your vaccine behavior?
     1. Tell me about your thought process when hearing your physicians’ recommendation.

IF “NO” TO HAVING CHILDREN ON QUESTIONNAIRE, SKIP TO III. PERTUSSIS KNOWLEDGE.

IF “YES” TO VACCINATING THEIR CHILDREN ON QUESTIONNAIRE:

**[Child]**

- 1. You filled in on the questionnaire that you have a child(ren). I’d like to hear about your experiences getting your child(ren) vaccinated.
  2. Why did you get your child(ren) vaccinated?
  3. What is it like bringing your child(ren) in to get vaccinated?
     1. Where do you find it easiest to get your vaccines?
     2. What part of the process of getting a vaccine would you change?
  4. How does the experience of getting your child(ren) vaccinated compare to getting vaccines for yourself?
     1. Did you have any concerns or questions about the vaccine(s) that you didn’t have when you were getting vaccinated?

IF “NO” TO VACCINATING THEIR CHILDREN ‘ON SCHEDULE’ ON QUESTIONNAIRE:

**[Child]**

- 1. You filled in on the questionnaire that you have a child(ren). I’d like to hear about your experiences getting your child(ren) vaccinated.
  2. What made you decide to not vaccinate/vaccinate on an alternative schedule?

**[VACCINATED, BUT OFF SCHEDULE]**

- 1. What is it like bringing your child(ren) in to get vaccinated?
     1. Where do you find it easiest to get your vaccines?
     2. What part of the process of getting a vaccine would you change?
  2. How does the experience of getting your child(ren) vaccinated compare to getting vaccines for yourself?
     1. Did you have any concerns or questions about the vaccine(s) that you didn’t have when you were getting vaccinated?

**[NOT VACCINATED]**

- 1. What has been the response from your child’s medical caregivers regarding your choice not to vaccinate?
     1. How do you feel about this feedback?

1. **Knowledge of Pertussis**
   1. What do you know about whooping cough?
      1. Is there anything else that you can think of that you know about this illness?
   2. Sometimes doctors tell us things about specific illnesses. What has your doctor told you about whooping cough?
   3. Besides your doctor, where else have you heard about whooping cough?
      1. Some people tell us that they get information about whooping cough from the internet, friends, and brochures at the health department. Can you think of any places where you have heard about whooping cough?
   4. Can you tell me about any experience you have had with the disease?
      1. I’d like to know more about anyone you know, in your family or community for example, who has had the disease.
   5. How likely do you think it is that you would get whooping cough?
      1. What about your children? [if applicable] .

Is there anything else you’d like to share with me about your experiences and ideas about vaccination or whooping cough?
